# Digital Health Paradox: International Policy Perspectives to Address Increased Health Inequalities for People Living With Disabilities

**DOI:** 10.2196/33819

**Published:** 2022-02-22

**Authors:** Robin van Kessel, Rok Hrzic, Ella O'Nuallain, Elizabeth Weir, Brian Li Han Wong, Michael Anderson, Simon Baron-Cohen, Elias Mossialos

**Affiliations:** 1 Department of International Health Care and Public Health Research Institute Maastricht University Maastricht Netherlands; 2 Studio Europa Maastricht University Maastricht Netherlands; 3 Global Health Workforce Network Youth Hub World Health Organization Geneva Switzerland; 4 Public Sector Strategy Team Deloitte Consulting Pty Ltd Sydney Australia; 5 Autism Research Center Department of Psychiatry University of Cambridge Cambridge United Kingdom; 6 The Lancet and Financial Times Commission on Governing Health Futures 2030: Growing up in a digital world Global Health Centre The Graduate Institute Geneva Switzerland; 7 Steering Committee European Public Health Association Digital Health Section Utrecht Netherlands; 8 Department of Health Policy London School of Economics and Political Science London United Kingdom; 9 Institute of Global Health Innovation Imperial College London London United Kingdom

**Keywords:** digital health, eHealth, health policy, health systems, disability, inclusion, digital technologies, people living with disabilities

## Abstract

The COVID-19 pandemic accelerated the uptake of digital health worldwide and highlighted many benefits of these innovations. However, it also stressed the magnitude of inequalities regarding accessing digital health. Using a scoping review, this article explores the potential benefits of digital technologies for the global population, with particular reference to people living with disabilities, using the autism community as a case study. We ultimately explore policies in Sweden, Australia, Canada, Estonia, the United Kingdom, and the United States to learn how policies can lay an inclusive foundation for digital health systems. We conclude that digital health ecosystems should be designed with health equity at the forefront to avoid deepening existing health inequalities. We call for a more sophisticated understanding of digital health literacy to better assess the readiness to adopt digital health innovations. Finally, people living with disabilities should be positioned at the center of digital health policy and innovations to ensure they are not left behind.

## The Digital Paradox as a Modern Wicked Problem

As foundational parts of society continue to digitalize [1], the risk of existing inequalities worsening cannot be overstated. Digital divides constitute a wicked challenge in European countries [2], especially considering how digital literacy and access to digital infrastructure differ across age, sex, socioeconomic and educational strata, place of residence, and disability status [2,3]. The COVID-19 pandemic has highlighted the benefit of advances in digital health technologies [4], which could be deployed at a large scale to reach population groups that are otherwise difficult to reach. This reflects the paradox of digital health that we are currently facing: the potential that digital health innovations hold can be transformational for delivering care to underserved population groups, but these groups are most likely to be excluded from the digital world through their sociodemographic characteristics [5].

One example of such groups is the autism community. Recent research suggests that autistic individuals have comparatively shorter life spans [6,7] and are more likely to experience nearly any chronic physical and mental health condition [8-10]. Despite greater health care utilization and expenditure [10-12], autistic individuals report lower quality health care, worse health care access, and poorer patient-provider communication [11,13-17]. In qualitative interviews, autistic adults expressed difficulties evaluating or describing their health, sensory sensitivities, executive function, body awareness, slow processing speed, stigmatization, and systemic barriers [13-16]. As a result, the autism community can be considered highly vulnerable to digital exclusion, not only because of the aforementioned challenges but also due to the poorer educational and employment outcomes associated with autism [18].

The European Commission recognizes in the Union of Equality: Strategy for the Rights of Persons with Disabilities 2021-2030 that accelerated digital transformation offers opportunities to remotely deliver high-quality care to people living with disabilities that is tailored to their needs [19]. However, the precise actions needed to develop an inclusive digital health system that benefits people living with disabilities instead of excluding them, as well as best practices, are undefined.

It is important to note that this article takes a holistic approach to digital health. In other words, digital health as a concept can refer to a technology, user experience, service, product, process, infrastructure of its own, or part of the ecological system of health services [20-24]. It is important to be mindful of all these potential framings of digital health to encourage a systems thinking approach to how digital health may be incorporated in the status quo of health care. While there is great potential in the deployment of digital health services, the digital paradox makes it painfully clear that the utility of digital health is proportional to how well it meets the health care needs of the person interacting with it.

In this article, we explore the ways in which digitalization affects the health sector, specifically looking at the distribution of information, social determinants of health, and access to digital infrastructure. We then highlight how these digital developments may affect people living with disabilities, using the autism community as a case study. Afterward, we showcase existing policy perspectives from Sweden, Australia, Canada, Estonia, the United Kingdom, the United States, Singapore, Japan, and South Korea that either facilitate or obstruct the creation of digital health systems accessible for people living with disabilities. Finally, we identify principles of digital health that can underpin and facilitate the safe and inclusive development of a digital health ecosystem. We believe this article may be of particular interest to policy makers, health practitioners, patient groups, health funders, social workers, and other stakeholders in the field of health care. Details on the methodology and the PRISMA (Preferred Reporting Items for Systematic Reviews and Meta-analyses) flowchart can be found in Multimedia Appendix 1.

## Impact of Digital Technologies on the Distribution of Information

While the relationship between digital technologies and health is nonlinear and multifaceted, digital technologies can facilitate the distribution and dissemination of public health information and action (eg, widespread broadcasts by the World Health Organization throughout the COVID-19 pandemic). Digital health can also enhance clinical and laboratory work by supporting administrative tasks, leaving more time for health care workers to spend with their patients [1,23]. This, however, requires a careful balance between increasing efficiencies through digital means and reducing the cognitive burden of frontline staff (explored later in this paper). Telemedicine can improve access to life-saving medical care. Soon, digital technology will augment clinical diagnostic processes, improve data collection and analysis, and provide targeted support in the form of precision medicine and precision public health [1,22,23,25].

On the other hand, health disinformation and misinformation can easily and rapidly be spread in a digital society through social media, contributing to the continued existence and strengthening of factually incorrect health beliefs [26]. A recent notable example of health misinformation has been the tremendous decline in vaccine confidence [27]. Machingaidze and Wiysonge [27] explain how digital technologies can be excellent tools for self-education, a key component of vaccination decision-making. However, they also present various challenges of using digital technologies at scale, many of which are present in high-income countries, including misinformation, incomplete information, and conflicting and complicated scientific information that may be difficult to understand [27].

## Digital Technologies and Social Determinants of Health

Digital technology can also further entrench established social determinants of health [24]. For example, wearable technologies hold great potential to equip the general public with the ability to proactively monitor and manage their health care, fitness, and aging [28,29], although this comes at a financial cost. Presently, this expense is borne by consumers with the ability to pay. This creates inequities in access to personalized health data, placing it at the fingertips of wealthier individuals and further widening health divides between socioeconomic groups. As such, when advocating for the widespread uptake of digital health among vulnerable groups, we must avoid constructing or fueling the paradigm of digital health as a pinnacle of health consumerism, as this would only expand the existing digital paradox [21]. A recent study in the United Kingdom highlights that access to free or affordable health care and individual behaviors and choices dominate the public perception of what impacts an individual’s health and that 24% of the UK population believes health is entirely the responsibility of the individual [30]. As a result, the belief that healthier choices are available to more resourceful population groups is commonly propagated, disregarding the potential and complex effects of social determinants of health.

Furthermore, economies of scale achieved through increased use will drive down the price of wearable technology [31]. A case study in rural India demonstrated the benefits of wearables for chronically underserved rural and remote populations, showing improved health outcomes, including decreased readmission and mortality rates, by monitoring health data and improving preventive care [32]. Furthermore, wearables reduced the need for costly transport and frequent doctor visits as a patient’s health could be observed at home and transmitted to their doctor in another locality [32]. This remote monitoring approach could prove hugely beneficial for people living with disabilities, as wearables are able to address various dimensions of disability, including physical, sensory, emotional, and intellectual conditions [33]. This approach could provide people living with disabilities with greater autonomy, augment their ability to live independently, and improve educational and employment outcomes [34].

Wearables have been shown to enable people to become informed about their health status and conditions, give patients a sense of agency over their health metrics, and improve health literacy [29,35]. Moreover, health care providers and governments can harness digital technology’s aggregated information and insights to improve the quality of diagnosis and broader population health [29,36]. As such, digital divides threaten the equitable access of health care and public health services and fundamentally disadvantage the strata of the population that cannot pilot digital technologies [3,22,24]. Therefore, measuring digital health literacy is critical as it will help evaluate the readiness of a health system to implement digital health innovations, design targeted educational tools before implementation, and avoid costly attempts that were certain to fail from the start [37].

## Differences in Access to Digital Technologies and Infrastructure

Considerable work has been done to measure the world population’s ability to access digital services in terms of available infrastructure and digital literacy [3,22,38]. Nevertheless, these efforts are often limited to a binary measure of internet access and an overly simplified set of digital skills [3,38]. As such, they fall short by failing to acknowledge complex or domain-specific skills required to maximize the benefits of digital medical services. Simultaneously, while digital technologies are frequently designed to be used at the individual level, a system-level approach is required to fully understand the breadth and depth of how digital technologies can shift or augment a (public) health system [23].

A crucial part of this system-level approach is the ability of frontline health care workers to engage with and effectively use digital (health) technologies and platforms to streamline their workflows. With digital health technologies, the technology must be user-friendly to relieve the cognitive burden of health workers so they can focus on providing medical care [39]. Fractured communication and different systems across facilities and departments are often significant reasons for increased cognitive load [39]. As such, by optimally managing and communicating information through standardized systems, the potentials of digital health could be maximized to achieve the most significant benefit for health professionals [39].

Nurses are well placed to drive the digital health literacy agenda and upskill their colleagues. Moreover, as nurses often act as the primary care providers for rural or vulnerable populations, they would benefit from developing the knowledge and skills to implement and use telehealth technologies [40]. Strengthening their ability to use digital health technologies should therefore be an integral part of education for nurses and other health professions. A multimodal training approach (ie, didactics, patient simulations, practice immersions, and project work) has enabled students to develop the skills required to embrace telehealth and be comfortable using it [40]. Simultaneously, medical training continues to focus heavily on the quantitative side while underserving the arts and humanities of medicine [41], inherently limiting the extent to which medical professionals can advocate for the influence of various social determinants on health. However, it is the social determinants that often play a multifaceted role in whether a patient is able to access digital health services. In order for digital health care to become a mainstream mode of health care, medical education needs to be rebalanced with adequate coverage of arts and humanities of medicine and social medicine to train future health professionals when it is appropriate to deploy digital services, especially as long as the digital divide remains as extensive as it is currently. Additionally, social prescribing (ie, referring people to a range of local, nonclinical services) is increasingly becoming a core role of clinicians [42]. This could be further ameliorated with specialized training programs to ensure they have the right tools and referral pathways on hand to deploy it effectively.

Virtual medicine has significantly changed the way health care professionals deliver care. The COVID-19 pandemic has accelerated the development of telehealth services to the point where they have become a viable pathway for remotely delivering accessible, cost-effective, and quality care to patients [40,43]. Although videoconferencing is increasingly regarded as an adequate substitute for face-to-face consultations, network coverage shortages and lack of digital access or literacy among doctors and patients have meant that a proportion of remote primary care still takes place via telephone [44,45]. This change in the modality of care provision is also expected to persist after the pandemic as users appreciate the convenience and accessibility afforded by telehealth services [40,46,47], especially in remote areas or for vulnerable populations who prefer or require access to health care closer to home. Digital health must, in turn, become more accessible, as must the incorporation of features that enable its acceptance and the reimbursement of this modality [46]. This is particularly important in resource-limited settings and low- and middle-income countries, where accessible pathways must be established. This will maximize the potential of telehealth to transform health care delivery for the wider global population [48].

## Digital Technologies, Health, and Disability

While people living with disabilities cannot be taken as a monolith, they are on average older, poorer, and less likely to have a regular health care provider (and thus less likely to seek health care regularly) [49]. They are also more likely to experience multiple co-occurring conditions [49]. These factors pose additional challenges for developing and integrating the infrastructure of telemedicine into the lives of people living with disabilities [50]. However, digital health platforms offer an opportunity for improved engagement with populations with complex health needs who may receive poorer quality health care services, including those on the neurodiversity spectrum.

Telemedicine appointments allow patients to interact with health care providers at home [40,48,51], limiting exposure to overwhelming and unknown sensory environments while also accommodating alternative forms of communication (eg, audio-based or chat-based), which may be preferred by autistic adults [11]. Telemedicine and digital health may provide an online record of health care appointments (past and future), diagnoses, and prescriptions that patients can view at any time [51], which may afford greater autonomy for autistic patients or caregivers in managing their health care. Finally, online access to health care records for patients—particularly those with complex care needs—can provide reminders for essential tasks (eg, attending future follow-up appointments, collecting prescriptions) and offer opportunities for health care records to be corrected in the event of miscommunication between patients and providers.

Systematic reviews of pediatric and adult populations found that services delivered via telehealth were equally effective or superior means of providing health care to people living with disabilities, including those on the autism spectrum [52-54]. Furthermore, other forms of digital health like wearable technologies may provide novel opportunities for remote monitoring of people living with disabilities in distress situations or promote healthy lifestyle choices (eg, diet, exercise, and sleep) [29,51]. These tend to be significantly worse among autistic adults relative to neurotypical people and are associated with excess risk of chronic cardiovascular conditions [55]. Although we have explored the potential advantages of integrating telemedicine into health care systems for autistic adults, these benefits may also apply to those with other disabilities and the general population. Thus, telemedicine may be an efficient and cost-effective means of beginning to close the gaps in equitable access to high-quality health care for all.

## Policy Perspectives and Practices

This section shows an overview of guidelines established in 3 countries to help facilitate the (digital) inclusion of people living with disabilities. Each set of principles shown in Textboxes 1, 2, and 3 [56-59] lays a foundation for a collaborative environment where all stakeholders are involved and empowered in the development and implementation of digital health, allowing for different vulnerable groups to be included during the development process to ensure their needs are met by the end product.

Sweden’s digital health initiatives have been ongoing for 15 years at the time of writing with its current Vision for eHealth 2025 being adopted in 2016 (Textbox 1) [56]. The Vision acknowledges the opportunities that digital health brings for improving the welfare and well-being of vulnerable communities. It states that for these opportunities to be actualized digital health should be nondiscriminatory and respond to the needs of different groups. This is possible given the inherent characteristic of digital health to be deployed at an individual level.

**Strategy for implementing Vision for eHealth 2025 in Sweden [57].**

Objectives:

- Involve the individual as cocreator
- Ensure that all relevant information is easily available when needed
- Guarantee that personal information is processed safely and securely
- Acknowledge that development and digital transformations go hand-in-hand

Fundamental principles:

- Regulations should (1) safeguard individual rights such as privacy, equality, patient safety, and accessibility; (2) enable the most optimal use of digital health innovations; and (3) stand the test of time and be specific enough to be applied
- Terminology and semantics should be used consistently across administrative systems to ensure and improve interoperability
- Acknowledging that a lot of standardization occurs at the European Union level, common and cross-sectoral standards (ie, procedures of application and development) are promoted to prevent national or sector-specific unique solutions

**National Disability Strategy–United Kingdom [58].**

The governmental approach to disability is guided by 5 principles:

- Ensuring equity and fairness: people living with disabilities will be empowered by advocating for fairness and equality in opportunities, experiences, and outcomes
- Considering disability from the start: inclusive and accessible approaches will be embedded in government functioning to mitigate the creation of excluding experiences for people living with disabilities
- Supporting independent living: initiatives that support all people living with disabilities to have choice, control, and autonomy in their life will be actively encouraged
- Increasing participation: a diverse group of people living with disabilities will be included in the design, development, and delivery of products, services, and policies
- Delivering joined-up responses: work will occur across organizational boundaries to improve data and evidence gathering to better understand the complex issues that affect people living with disabilities

**Accessible Canada Act [59].**

The purpose of this legislation is to benefit all persons, especially people living with disabilities, by removing existing barriers and preventing new barriers within Canada on or before January 1, 2040. It sets out 7 principles to guide the transformation process:

- Every person must be treated with dignity regardless of their disabilities
- Every person must have the same opportunity to make for themselves the lives that they are able and wish to have regardless of their disabilities
- Every person must have barrier-free access to full and equal participation in society, regardless of their disabilities
- Every person must have meaningful options and be free to make their own choices, with support if they desire, regardless of their disabilities
- Laws, policies, programs, services, and structures must take into account the disabilities of persons, the different ways that persons interact with their environments, and the multiple and intersecting forms of marginalization and discrimination faced by persons
- People living with disabilities must be involved in the development and design of laws, policies, programs, services, and structures
- The development and revision of accessibility standards and the making of regulations must be done with the objective of achieving the highest level of accessibility for people living with disabilities

This sentiment also arose from the United Kingdom Disability Survey 2021, which indicates that “44% of [people living with disabilities] who received [online] formal care said it made them feel more in control or much more in control of their lives” [58]. For this transformation to occur sustainably, strong principles are required to guide and facilitate the transformation process.

In terms of operationalizing the fundamental principles in Textboxes 1, 2, and 3, Australia and Canada showcase good policy practices. Australia adopted the Digital Transformation Strategy 2018-2025 in 2018, which is geared toward the digital transition of government services [60]. The strategy includes the Digital Service Standard criteria, which extensively outlines how developers and innovators can ensure that their services are usable by every person who needs them [61]. The guidelines distinguish 4 phases of progress (discovery, alpha, beta, and live), each containing between 4 and 15 requirements that need to be met before the next phase can begin. During the first 3 phases, innovators must prove that people from different backgrounds and people living with disabilities were involved in the development process and that the innovation is accessible for these groups. While the guidelines were designed for innovation in government services, the criteria are good practice guidelines for inclusive digital innovation in every sector, including health care. These guidelines can be further augmented by making funding contingent on adherence to such guidelines. The Accessible Technology Program in Canada is an example of this. Even though the program description does not offer extensive guidelines like the ones set out in Australia, its overarching aim is similar: “to develop innovative assistive and adaptive digital devices and technologies for persons with disabilities” [62].

Alternatively, the United Kingdom provides an extensive list of organizations that give support, advice, or information for each stage of the development life cycle for digital health and care products [63].

When considering active citizen engagement, we must consider what meaningful or equitable engagement looks like in health research. Literature on various frameworks for patient or citizen engagement is rich [64-67], although there is a common consensus among these studies that the frameworks themselves are lackluster and no specific research on the involvement of people living with disabilities or vulnerable groups has been carried out. As such, we are not in the position to establish concrete guidelines for meaningfully including people living with disabilities in the co-design process. That said, there is a consensus that meaningful engagement adheres to a number of principles (eg, the INVVOLVE principles: Invest in co-design; Needs assessment; Vision roles, responsibilities, and rewards; Validate participants; Organize interaction carefully; Lead the engagement; Value patient time and input; Evaluate and report [67]). When determining a method for meaningful engagement of people living with disabilities, it is good practice to consider the ability and preferences of the participants [68]. For instance, workshops can be a meaningful way to invite and interact with a representative group of people living with disabilities throughout the design process [69] but may only attract certain clusters that can handle the sensory or cognitive input paired with attending workshops. Ratwani and colleagues [70] further elaborate on some good and bad practices regarding user-centered design processes in the development of electronic health records that can be particularly relevant for informing co-design methods for people living with disabilities.

Estonia is currently regarded as the frontier of how an integrated digital health system can function [71,72]. Estonia offers citizens the ability to access diagnostic services, consultations, prescription refills, and referrals online. The digital health system is built on the basis of an information society, which was initiated in 1992. Having regained independence from the Soviet Union in 1990, Estonia was able to rebuild and develop a society pointed toward the Digital Age without the remnants of past times. In contrast, most Western countries are run by long-established governments and sometimes even longer established ideologies, which may constrain the adoption of transformational innovations that significantly challenge the status quo [73].

For example, health care coverage and insurance, including telemedicine access, remains disjointed and variable for people living with disabilities in the United States. In 1990, the Americans With Disabilities Act (ADA) was passed to protect people living with disabilities against discrimination, specifically requiring that health care entities provide full and equal access for people living with disabilities. However, until the passage of the Affordable Care Act (colloquially known as Obamacare) in 2010, insurers could discriminate against people living with disabilities by charging higher premiums or denying health care coverage by capitalizing on an exemption in the ADA [74]. As the United States does not offer universal health care or health insurance schemes, service access for people living with disabilities presently depends on insurance type: Medicaid (37.7%), Medicare (27.1%), private insurance (36.1%), military benefits (6.0%), and uninsured (8.5%) [75], in turn providing patchwork telemedicine coverage. This is particularly true for Medicaid (the largest single provider of health insurance coverage for people living with disabilities), as each state runs their Medicaid system independently, individually interpreting federal mandates (like the ADA) and definitions of telemedicine. As of 2021, both Medicare and Medicaid universally offer some access to telemedicine services [76]; however, these programs are limited based on location, plan type, service type, provider type, and provider licensing state, and prescriptions may not be allowed via telemedicine. Further, only 10 state-run Medicaid programs offer all 3 major types of telemedicine to enrollees: live conferencing, remote patient monitoring, and store-and-forward electronic health records [76]. As it stands, this system (or lack thereof) leaves people living with disabilities either unable to access a wide range of telemedicine services or forced to reckon with significant system-level barriers, including deciphering individual insurer policies (and sometimes individual state insurance policies) to determine which types of telemedicine coverage can be reimbursed, much less whether their specific provider will be covered. Finally, it cannot be overstated how access issues compound when dealing with intersectional populations (including those experiencing poverty, homelessness, racism, and sexism along with disabilities), in turn providing greater disparities in health care coverage as well as greater opportunities for closing the gap via telemedicine.

Policy searches were also performed for South Korea, Japan, and Singapore. However, it appeared that digital transformation strategies were not yet developed in these countries, which is not only a contrast to the Western world but also to the fact that these countries are perceived as leaders in the field of digital transformation [77].

## Limitations

Before accurate recommendations for policy and practice can be established, the circumstances under which this article was written need to be considered. The findings of this article should be interpreted as scoping and are not definitive due to the unsystematic nature of data collection. Our aim is to inspire more thorough research and initial/preliminary action in the discussed topics. Additionally, the quality of included sources has not been assessed, which reinforces the need to interpret the results carefully.

Moreover, the author team consists almost exclusively of young professionals who grew up during a period when digital technologies flourished and had access to digital technologies during their formative years. The author team also exclusively consists of people with a Western background (European/North American). Consequently, the values, interpretations, opinions, and recommendations in this piece—while rooted in scientific evidence—may be considered progressive and transformational when presented to population groups that have different relationships with digital technologies. That said, the demographic makeup of the author team can also be considered a strength of the article, as youth and young professionals have a strong stake in the future development of the health system.

## Principles for Policy, Research, and Practice

The policy perspectives above point toward a strong need to work collaboratively across sectors. Technological, social, organizational, and political innovations that fundamentally reimagine health care delivery must be embraced with the aim to establish a new social contract that is fit for purpose for delivering high-quality digital health care to people living with disabilities [78,79]. At the same time, these policy perspectives highlight how digital health is currently framed in policy as a technology or a service more so than anything else. It is framed to be used by a consumer or a patient rather than be delivered by a health professional.

For effective implementation of digital (health) technologies, legal challenges, security breaches, regulatory concerns, and industry barriers must be addressed. Existing legislation, regulations, and policies have largely been written for face-to-face health care delivery, and security regulations vary widely across (and even within) territories [43,44]. When considering the developmental trajectory of Estonia in the field of digital health, it becomes evident that a collaboration of public and private stakeholders, including national governments, patient groups, international governmental and nongovernmental organizations, citizen representatives, health care representatives, and social sectors, must convene to consider whether the values underpinning existing health care structure are still fit for purpose. Subsequently, this coalition should move to develop a high-level consensus on definitions, boundaries, protocols, monitoring, evaluation, and data privacy that is needed to meet the rapid advancement of digital health [47]. Specifically, they should focus on reimbursement of digital health care, privacy/cybersecurity, liability, licensure, and technology access, while ensuring that all countries participating in this coalition meet an internationally recognized minimum standard [80]. International and national policy makers need to amend the existing policy framework to support and enable new ways of working driven by digital health [57,60]. In the interim, given that many professional liability policies currently exclude digital health from coverage, health care practitioners are advised to acquire additional coverage to ensure protection from liability issues [43].

Designing an ecosystem of digital health technologies with health equity in mind is imperative to avoid deepening health inequalities [81]. In other words, the burden of implementing digital health innovations should not fall on citizens by focusing solely on upskilling after digital health tools are developed. This applies doubly when aiming to reach people living with disabilities and other groups that are frequently digitally excluded and may garner unique benefits from these services. Following the Swedish and Australian examples, policy makers can draft policies that include vulnerable groups as integral parts of the co-design process. Additionally, funding agencies can follow the Canadian example and require that people from different backgrounds, including people living with disabilities, are included at every stage to get funding for a project.

Future research should work toward adopting a broader and more sophisticated understanding of digital health literacy (for instance, the transactional model of eHealth literacy [82]). Subsequently, digital health research should meaningfully engage with vulnerable population groups to ensure that new digital tools meet their individual needs [57,60,62,83]. Specifically, a new relationship between qualitative and quantitative research fields needs to be established that considers qualitative and quantitative research complementary to each other [84]. Related to this is the need for software developers to partner with user experience and user interface experts in designing digital health technologies accessible to everyone [85] and reduce rather than add to the workload of health care professionals. The relationship between qualitative and quantitative research may also need to be reconsidered as new data streams become available through digital health innovations.

In summary, there is a role for national governments, actors from private sectors (eg, telecommunication, technology), international/global governing bodies (eg, World Health Organization, Organisation for Economic Co-operation and Development), and other stakeholders to collaboratively create a regulatory framework that ensures a high level of quality in digital health care through independent evaluation and certification [20]. The task of quality assurance should not fall to the individual. In other words, a systems thinking approach involving as many stakeholders as possible is paramount to safely and sustainably implementing digital transformations of this magnitude. This approach should be complemented by novel forms of collaborative governance and leadership that are goal-oriented, embrace risk-taking, and derive value from improved patient and societal outcomes rather than monetary gain [78]. The good practices and recommendations in this article have been condensed into domain-specific principles shown in Textbox 4. These principles are designed to facilitate the initial stages of development of a digital health ecosystem that is accessible to all.

**Principles focused on policy, practice, governance, and education for the sustainable integration of digital health innovations.**

Health policy:

- Digital health is framed as a multidimensional concept that can refer to a technology, user experience, service, product, process, infrastructure of its own, or part of the ecological system of health services
- Digital health care services are a viable way to deliver health care to underserved populations that is comparable in quality to analog health care
- Health data, a major driver of digital health care, is stored safely and securely with the patient having autonomy over with whom the information is shared
- People living with disabilities and vulnerable groups are core subjects upon which digital health-related policy is built

Health care design and delivery:

- The design and development of digital health services are in close collaboration with people living with disabilities and other vulnerable population groups
- The involvement of people living with disabilities and other vulnerable groups in co-designing digital health services is systematic, purposeful, and equitable
- Clear agreements regarding expectations, input, and reward are made before the design process starts
- Digital health services are released and monitored collaboratively with people living with disabilities and other vulnerable groups

Governance:

- Governance approaches within health care embrace risk-taking, learn to function based on incomplete information, and assess value by outcome measures instead of monetary costs
- Collaborative governance is a central tenet in the governing process of health care that drives the other fields of policy making, health care design and delivery, and education

Education:

- A baseline course on the social determinants of health and their relevance to individual health is included in educational curricula of primary or secondary education
- A more in-depth course on the social determinants of health or social medicine is included in the educational curricula of all health professions
- Digital health literacy is a skill that needs to be maintained and updated intermittently to consistently engage with digital health services over time

## Conclusions

Digital technology is fundamentally reshaping the way health care is delivered. The current scope of its use is not fully living up to its potential. The COVID-19 pandemic saw digital health catapulted into widespread use as virtual delivery became a priority for many health services worldwide. However, the pandemic also emphasized that digital health in its current form exacerbates health inequalities. At greatest risk of digital exclusion are older people, people in rural and remote areas, and people living with disabilities.

While the futuristic use of digital technologies is auspicious, we must allow both the policy landscapes and global digital health literacy levels to catch up with the rapid advances in technology [86]. We must ensure that digital technologies are used equitably and do not become an exclusive domain of high-income countries or populations. People living with disabilities and other vulnerable groups must be put at the center of digital health development on a global scale. Ultimately, achieving equitable access to digital health will significantly benefit the health and well-being of the wider population, especially vulnerable groups, and go a long way in reaching the Agenda for Sustainable Development 2030.

## Appendix

Multimedia Appendix 1 Supplementary materials.

## Abbreviations

ADA: Americans With Disabilities Act
INVVOLVE principles: Invest in co-design; Needs assessment; Vision roles, responsibilities, and rewards; Validate participants; Organize interaction carefully; Lead the engagement; Value patient time and input; Evaluate and report
NIHR: National Institute for Health Research
PRISMA: Preferred Reporting Items for Systematic Reviews and Meta-analyses
SFARI: Simons Foundation Autism Research Initiative
