# Supplementary material for: Digital Health Paradox: International Policy Perspectives to Address Increased Health Inequalities for People Living With Disabilities
Source: J Med Internet Res. 2022 Feb 22;24(2):e33819. doi: 10.2196/33819 (PMC8905475; doi:10.2196/33819)
Supplement: Multimedia Appendix 1 [file jmir_v24i2e33819_app1.docx]

**Multimedia Appendix 1. Supplementary materials.**

Van Kessel R, Hrzic R, O’Nuallain E, Weir E, Wong BLH, Anderson M, Baron Cohen S, Mossialos E. The digital health paradox: international policy perspectives to address the increased health inequalities for people living with disabilities.

**Methods**

Information on the methodology used in this study.

**Study Design**

A scoping review is particularly useful when a body of literature has a complex and heterogeneous nature and is previously under-researched [[1]](https://www.zotero.org/google-docs/?zaDOj7). It is also widely used to investigate emerging fields in order to assess the extent, type, and nature of the evidence that is available [[1,2]](https://www.zotero.org/google-docs/?ragvvi). Even though the digitalisation of society has been ongoing for approximately a decade, it has substantially accelerated and received more attention during the COVID-19 pandemic. A scoping review is a well-suited methodology to explore this emerging field and summarize and disseminate research findings, as well as to identify action points for future work [[1,2]](https://www.zotero.org/google-docs/?fliNrU).

**Information Sources and Search Strategy**

Scientific articles were identified through three scientific databases: PubMed, Google Scholar (first 200 hits) and Web of Science. Scientific databases were searched based on the following keywords: “digital health”, “mobile health”, “mhealth”, “ehealth”, “telemedicine”, “disability”, and “disabled”. From these keywords, the following search query was established: "(digital health OR mobile health OR mhealth OR ehealth OR telemedicine)" and "(disabilit* OR disabl*)". This query was adjusted for PubMed and Web of Science (eTable 1). Gray literature was also searched for relevant articles.

Policy documents and strategies from Sweden, Canada, Australia, the United Kingdom, the United States, South Korea, Singapore, and Japan were identified through national policy databases (eTable 2) and Google searches. We utilised a combination of the following keywords for the identification of policies: “digital health”, “digital”, “ehealth”, “e-health”, “disability”, “strategy”, “law”, “policy”, “regulation”, and “directive”. These keywords were paired with every country that was considered for analysis in the Google searches: (“Sweden” OR “Swedish), “Canad*”, “Australia*”, (“United Kingdom” OR “UK”), (“United States” OR “US” OR “USA” OR “America*”), (“Korea*” OR “South Korea*”), “Singapore*”, and “Japan*”. Keywords were translated into the respective native languages using Google Translate, which is useful for the translation of loose words and shallow terms [[3]](https://www.zotero.org/google-docs/?r7bX2G).

**Eligibility Criteria and Data Extraction**

Scientific articles using any study design were considered eligible for inclusion if they were published in peer-reviewed journals from 2014 to 2021. For policy documents, a time limit of three years was used (2018-2021) to ensure the policies discussed in this article are still in effect and relevant. In terms of content, scientific articles and policies were included if they discussed the application of digital health for people living with disabilities. In case people living with disabilities were not mentioned, a secondary search was done to determine if vulnerable groups were covered. If this was the case, the article was also included. Given the nature of a scoping review, the inclusion criteria is kept broad to ensure all aspects and dimensions of digital health are covered.

The academic search was executed by one author (RVK) and the search results were split equally across four authors (RVK, RH, E'ON, and EW) for the data extraction due to time constraints. The policy search and policy data extraction was entirely performed by one author (RVK). The synthesis of both searches were reviewed by four authors (BLHW, MA, SBC, and EM) to identify missing information and ensure completion.

The results of the search strategy are presented in a PRISMA flowchart in eFigure 1. Policy documents included in the narrative synthesis were organised by country. No quality assessment of included studies was performed.

**Table S1.** Search queries for individual scientific databases.

|  | Query |
| --- | --- |
| PubMed | (digital health[MeSH Terms] OR mobile health[MeSH Terms] OR mhealth[MeSH Terms] OR ehealth[MeSH Terms] OR telemedicine[MeSH Terms]) AND (disabilit*[MeSH Terms] OR disabl*[MeSH Terms]) |
| Web of Science | (ALL=(digital health OR mobile health OR mhealth OR ehealth OR telemedicine)) AND (ALL=(disabilit* OR disabl*)) |
| Google Scholar | (“digital health” OR “mobile health” OR “mhealth” OR “ehealth” OR “telemedicine”) AND (“disabilit*” OR “disabl*”) |

**Table S2.** Policy databases used per website.

| **Country** | **Policy Database** |
| --- | --- |
| Sweden | <https://www.government.se> |
| Canada | <https://laws-lois.justice.gc.ca/eng/> |
| Australia | <https://www.dta.gov.au> |
| United Kingdom | <https://www.legislation.gov.uk/browse/uk> |
| Estonia | <https://www.riigiteataja.ee/index.html> |
| United States | <https://www.usa.gov/laws-and-regs> |
| Singapore | <https://sso.agc.gov.sg/> |
| Japan | <https://www.meti.go.jp> |
| South Korea | <https://elaw.klri.re.kr/eng_service/main.do> |

**Figure S1.** A PRISMA flowchart presenting the data selection process.


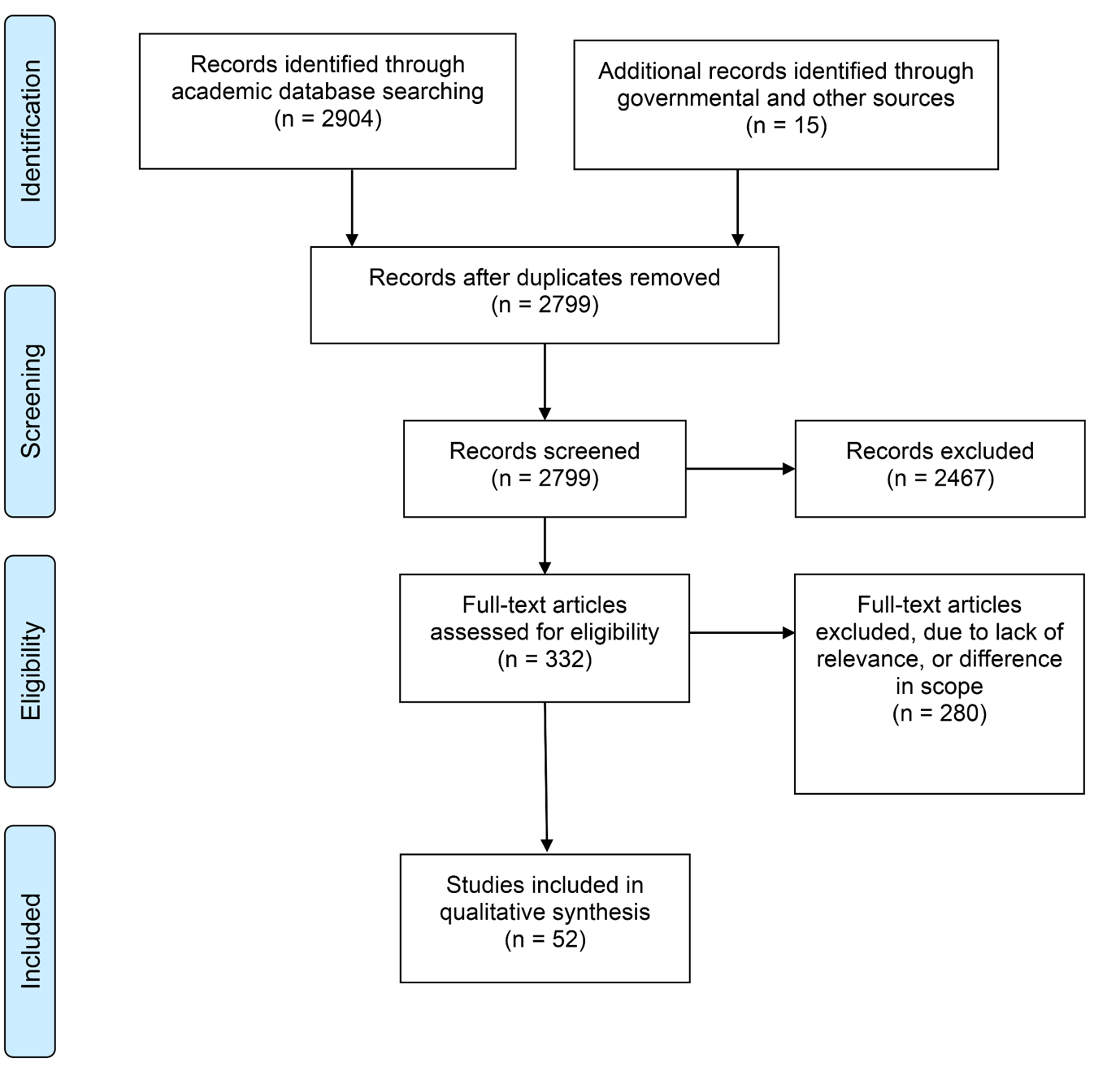


References

1. Arksey H, O’Malley L. Scoping studies: towards a methodological framework. Int J Soc Res Methodol. 2005 Feb 1;8(1):19–32.
2. Levac D, Colquhoun H, O’Brien KK. Scoping studies: advancing the methodology. Implement Sci. 2010 Sep 20;5(1):69.
3. Groves M, Mundt K. Friend or foe? Google Translate in language for academic purposes. Engl Specif Purp. 2015 Jan 1;37:112–21.
